# Orbital cellulitis and brain abscess – rare complications of maxillo-spheno-ethmoidal rhinosinusitis

**DOI:** 10.22336/rjo.2017.25

**Published:** 2017

**Authors:** Farah Constantin, Patricia-Alexandra Niculescu, Oana Petre, Daniel Balasa, Alexandru Tunas, Ioana Rusu, Mihai Lupascu, Cristiana Orodel

**Affiliations:** * Department of Ophthalmology, Clinical Emergency County Hospital, Constanta, Romania; ** Department of Neurosurgery, Clinical Emergency County Hospital, Constanta, Romania; *** ENT Department, Clinical Emergency County Hospital, Constanta, Romania

**Keywords:** orbital cellulitis, rhinosinusitis, brain abscess, E. Coli

## Abstract

Sinus infections can be complicated by ocular infections and, in late phases, by brain parenchyma infection.

The article debates the case of a 12-year-old patient suffering from paucisymptomatic maxillo-spheno-ethmoidal rhinosinusitis, which was later complicated by orbital cellulitis, ending with the development of a brain abscess.

The treatment is complex, initially targeting the source of the infection through draining the collection by middle maxillary antrostomy and anterior posterior ethmoidectomy, then the ablation of the brain abscess and postoperatively with prolonged massive antibiotherapy.

**Abbreviation:** URI = upper respiratory infection, CT = computer tomography, MRI = magnetic resonance imaging, BA = brain abscess, VAS = visual scale of pain, ENT = ear, nose, throat, RE VA = right eye visual acuity, RE = right eye, CSF = cerebrospinal fluid

## Introduction

Rhinosinusitis is becoming an increasingly common diagnosis in children and can be a frustrating problem. More young children are attending day care centers, increasing the transmission of URIs. Environmental pollutants and allergens predispose children to rhinosinusitis [**1**].

Normal paranasal physiology requires patency of the ostia, normal mucociliary clearance, and normal secretions. When any one of these changes appears, ostial obstruction, retention of secretions and infection can occur [**1**].

Adenoid hyperplasia and infections may predispose a child to rhinosinusitis. Large or infected adenoids may produce nasal obstruction and symptoms of rhinosinusitis. The adenoids may serve as a reservoir for pathogenic bacteria [**1**].

The incidence of complications from pediatric rhinosinusitis is greater than in adults [**1**].

According to Sarafoleanu Codrut, among the contiguity complications of sinusitis, the orbital cellulitis and the brain abscess can be frequently found located in the frontal lobe [**2**].

According to M. Yanoff & J. S. Duker, the orbit and the adnexa of the eye are important locations for primary and secondary pathologies because various types of tissue, such as vascular, bone, nervous, muscle and glandular are present at that level. The tumors and inflammations can secondarily invade the orbit from periorbital regions, such as the paranasal sinuses, the eyelids, and the intracranial compartments [**3**].

Orbital cellulitis is an acute serous and diffuse inflammation of the cellular-adipose tissue of the orbit. The cause can be exogenous (posttraumatic) or through propagation of a nearby inflammatory process [**4**].

Orbital complications are the most common problem associated with rhinosinusitis and have the highest incidence in children [**1**]. The source of infections of the orbit is usually the ethmoid sinuses, but may also be the frontal and maxillary sinuses. The routes of spread are direct, hematogenous, by arterial or venous thrombophlebitis, and lymphatic. The medial orbital wall is an ineffective barrier against the spread of infection because it has natural dehiscences, suture lines, and thin bone [**1**].

The most common bacteria are Staphylococcus and Streptococcus in adults, and in children, Haemophilus Influenzae. E. Coli and Pseudomonas are the rarest [**3**].

Brain abscess is defined as a focal intracranial infection that is initiated as an area of cerebritis and evolves into a collection of pus surrounded by a vascularized capsule. Given their location, the approach to brain abscesses often presents diagnostic and therapeutic challenges [**5**].

Brain abscess may arise from hematogenous spread, contiguous spread, or direct trauma [**6**].

The clinical manifestations of brain abscess may run the gamut from indolent to fulminant; most are related to the size and location of the space-occupying lesion within the brain and the virulence of the infecting organism [**5**].

The classic triad of fever, headache, and focal deficit is present in less than 50%. Headache, usually dull and poorly localized is present in more than 70% of the cases, and is so nonspecific as to be a potential cause of diagnostic delays [**7**].

## Case report

A 12-year-old patient presented herself in the Department of Ophthalmology for a pronounced inflammatory eyelid edema on the right eye, frontal and occipital moderate headache, without exophthalmia, without fever.

Ophthalmological examination in internment: RE eyelid inflammatory marked edema with sudden onset, closed eyelid slot, local induration, local pain and calor, VAS 4/ 10, especially on palpation, RE VA = 1. The ENT examination at internment: normal. Neurological examination in internment: normal.

**Fig. 1:**
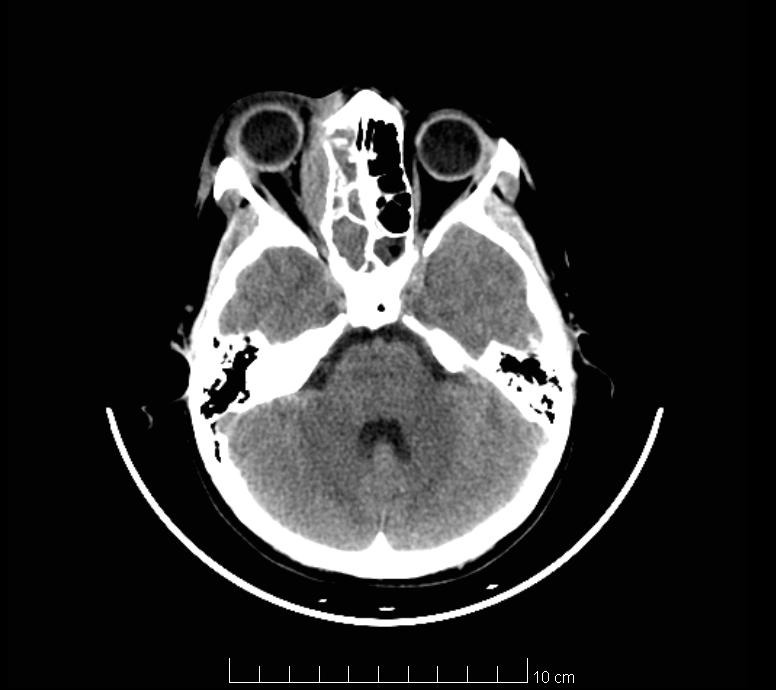
Preoperative Head CT-Scan (blue arrow – orbital cellulitis and osteitis, red arrow - sinusitis)

**Fig. 2:**
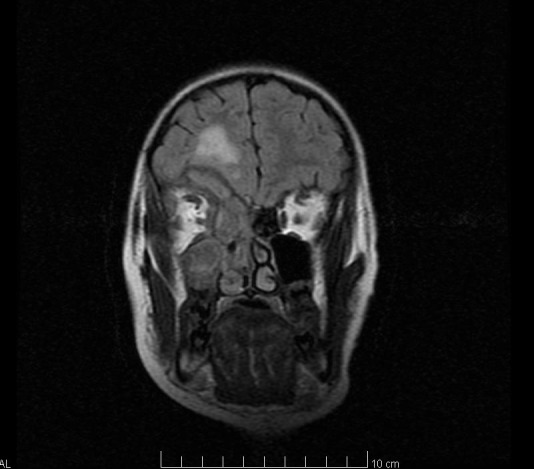
Preoperative Head MRI (blue arrow - orbital cellulitis, red arrow - sinusitis, yellow arrow - brain abscess)

Imaging tests (brain CT and brain MRI) highlight right frontal lobe abscess (**Fig. 2** – yellow arrow), medial orbital wall osteitis – right orbit (**Fig. 1** – blue arrow, **Fig. 2** – blue arrow), maxillary sinusitis, right ethmoidal and sphenoidal sinusitis (**Fig. 1** – red arrow, **Fig. 2** – red arrow). 

The course of treatment was decided while following the results of the imaging tests. This first included the ENT surgical treatment, in order to progressively clear the infection sources, then, secondly, the neurosurgical treatment.

The resection of the inflamed masses at the middle meatus was performed after endoscopically draining the collection through middle maxillary antrostomy, anterior posterior ethmoidectomy. Adenoidectomy was performed on the cavum. Wanted result: dissection of the maxillary and ethmoidal sinuses.

After 24 hours, the neurosurgical intervention was performed: frontal basal craniotomy with the excision of a grey-yellow purulent-granulomatous collection. Bacteriological test: E. Coli. 

**Fig. 3:**
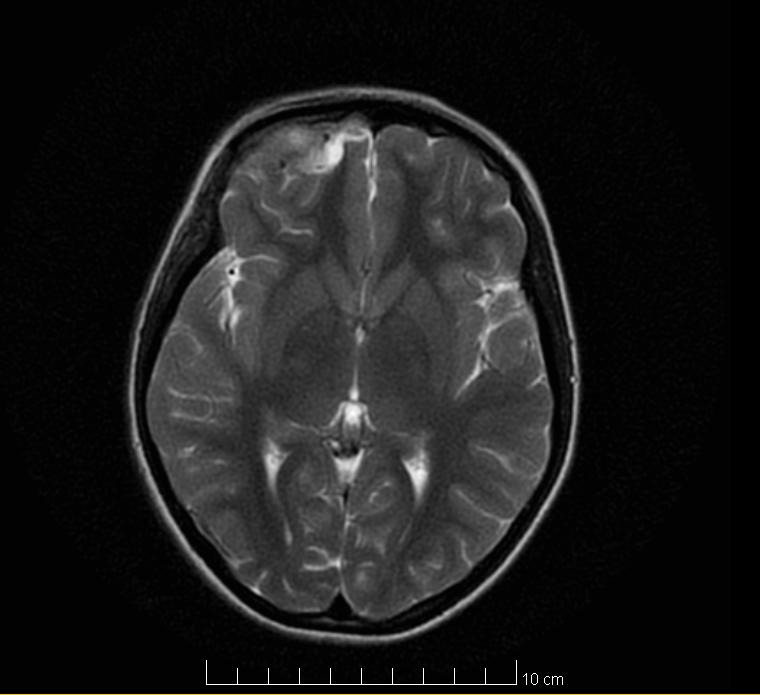
Postoperative Head MRI (yellow arrow - BA remission)

**Fig. 4:**
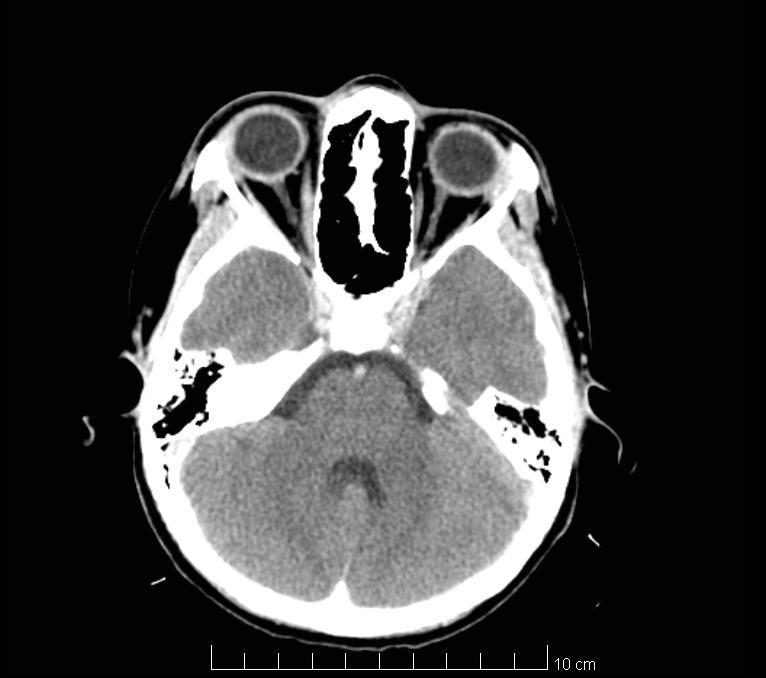
Postoperative Head CT-Scan (blue arrow - orbital cellulitis and osteitis remission; red arrow - sinusitis remission)

The revaluation imaging tests (brain CT and brain MRI) highlighted the remission of the intracranial purulent collection (**Fig. 3**), remission of the inflammatory phenomenon on the right orbit, with the recreation of bone structure on the lateral wall of the right ethmoidal lateral mass and the superior wall of the right orbit (**Fig. 4**).

Postoperatively, under intensive antibiotic treatment (Vancomycin, Meropenem, Gentamicin, Ceftriaxone, Cortisone, vitamins), clinical evolution in favorable, the patient continued with antibiotherapy in an Infectious Diseases Department until healing, according to the international protocols.

N.B.: After surgery, the antibiotherapy lasted 8 weeks.

## Discussion

The probability diagnostic pleaded for preseptal cellulitis, infirmed by the CT test, which highlighted orbit cellulitis in association with maxilla-spheno-ethmoidal rhinosinusitis and brain abscess.

Although symptoms in sinusitis are often associated with fever and purulent nasal discharge, in our case, the patient was paucisymptomatic.

A contiguity infection from the sinus to the intracranial cavity – CSF, has a higher contamination probability. The CSF composition (glucose, proteins, water) and a constant 37oC temperature is an excellent breeding ground for bacteria cultures: 1 ml of CSF contains maximum 5 white elements, compared to 1 ml of blood that has 5.000–10.000 white elements, which means that bacteria do not have to be extremely virulent to create meningocerebral infections, meaning that less virulent or saprophytes, can develop fast once they reach the levels of CSF. Also, in encapsulated infections, the bacteria resist even more because of the capsule, even when being incompletely formed, which massively decreases antibiotic penetration, which already penetrate the hematoencephalic barrier very slowly.

The enteric gram-negative bacilli (e.g. E. Coli) are isolated in 23-33% of the patients with brain abscess, through fronto-ethmoidal or sphenoidal sinusitis [**8**].

In a rat model of experimental brain abscess, E. Coli failed to cause infection in the skin, but abscess formation in brain tissue was induced [**5**].

The case particularity consists in the ophthalmologic debut of a very virulent pathology that had very few symptoms and clinical signs.

Without further investigation, the evolution of this case would have been unfavorable, even bad.

The attention given even to an inflammatory eyelid edema without exophthalmia can prevent the delay of providing an etiological diagnostic.

## Conclusions

Surgical treatment (maxillary sinus and ethmoidal dissection and brain abscess excision) represents an important part of the therapeutical strategy of these lesions.

According to the abibiogram, prolonged (8 weeks in this case) antibiotic treatment has offered the possibility of a full healing of both the sinus and cerebral regions and of the orbital osteitis.

The antibiotics used had a maximum penetrability both for CSF and bone tissue levels.
